# State-of-the-Art Production Chains for Peas, Beans and Chickpeas—Valorization of Agro-Industrial Residues and Applications of Derived Extracts

**DOI:** 10.3390/molecules25061383

**Published:** 2020-03-18

**Authors:** Annalisa Tassoni, Tullia Tedeschi, Chiara Zurlini, Ilaria Maria Cigognini, Janos-Istvan Petrusan, Óscar Rodríguez, Simona Neri, Annamaria Celli, Laura Sisti, Patrizia Cinelli, Francesca Signori, Georgios Tsatsos, Marika Bondi, Stefanie Verstringe, Geert Bruggerman, Philippe F. X. Corvini

**Affiliations:** 1 Department of Biological Geological and Environmental Sciences, University of Bologna, Via Irnerio 42, 40126 Bologna, Italy; 2 Department of Food and Drug, University of Parma, Parco Area delle Scienze 27/A, 43124 Parma, Italy; tullia.tedeschi@unipr.it; 3 Experimental Station for Food Preservation Industry, Viale F. Tanara, 31/A, 43121 Parma, Italy; chiara.zurlini@ssica.it (C.Z.); ilaria.cigognini.cococo@ssica.it (I.M.C.); 4 Institut für Getreideverarbeitung GmbH, Arthur-Scheunert Allee 40/41, 14558 Nuthetal, Germany; J.Petrusan@dil-ev.de; 5 IRIS Technology Group, Avda. C. F. Gauss 11, 08860 Castelldefels, Spainsneri@iris.cat (S.N.); 6 Department of Civil, Chemical, Environmental and Materials Engineering, University of Bologna, Via Terracini 28, 40138 Bologna, Italy; annamaria.celli@unibo.it (A.C.); laura.sisti@unibo.it (L.S.); 7 Department of Civil and Industrial Engineering, University of Pisa, Largo Lucio Lazzarino 2, 56126 Pisa, Italy; patrizia.cinelli@unipi.it (P.C.); francesca.signori@unipi.it (F.S.); 8 National Interuniversity Consortium of Materials Science and Technology, Via G. Giusti 9, 50121 Firenze, Italy; 9 Cosmetic Tsatsos Georgios, Ioannou Metaxa 56, 19441 Koropi, Greece; g.tsatsos@cosmetic.com.gr; 10 Conserve Italia Scarl, Via Paolo Poggi 11, 40068 San Lazzaro di Savena (BO), Italy; mbondi@ccci.it; 11 Nutritional Solutions Division, Nutrition Sciences NV, Booiebos 5, 9031 Drongen, Belgium; stefanie.verstringe@nusciencegroup.com (S.V.); geert.bruggeman@nusciencegroup.com (G.B.); 12 Institute for Ecopreneurship, School of Life Sciences, Fachhochschule Nordwestschweiz, Hofackerstrasse 30, CH-4132 Muttenz, Switzerland; philippe.corvini@fhnw.ch

**Keywords:** agro-industrial by-products, biowaste, biomass, fibers, legumes, plant proteins

## Abstract

The world is confronted with the depletion of natural resources due to their unsustainable use and the increasing size of populations. In this context, the efficient use of by-products, residues and wastes generated from agro-industrial and food processing opens the perspective for a wide range of benefits. In particular, legume residues are produced yearly in very large amounts and may represent an interesting source of plant proteins that contribute to satisfying the steadily increasing global protein demand. Innovative biorefinery extraction cascades may also enable the recovery of further bioactive molecules and fibers from these insufficiently tapped biomass streams. This review article gives a summary of the potential for the valorization of legume residual streams resulting from agro-industrial processing and more particularly for pea, green bean and chickpea by-products/wastes. Valuable information on the annual production volumes, geographical origin and state-of-the-art technologies for the extraction of proteins, fibers and other bioactive molecules from this source of biomass, is exhaustively listed and discussed. Finally, promising applications, already using the recovered fractions from pea, bean and chickpea residues for the formulation of feed, food, cosmetic and packaging products, are listed and discussed.

## 1. Introduction

In recent years, the management of agro-industrial and food processing by-products, residual matter and waste has arisen considerable interest from farmers, food producers, retailers and consumers. The food and food ingredient industrial production is associated with the generation of large, and sometimes unavoidable, by-products and waste streams; about 38% of the residues wastes are generated during food processing [1]. According to the Food and Agriculture Organization of the United Nations (FAO), about 1.3 billion tons of food is lost or wasted per year, along the whole production chain starting at the production stage and ending at the consumer level. Among all food commodities, fruit and vegetables (such as legumes) are the largest food waste contributor representing 44% of the global food waste, roots and tubers contributing by 20% and cereal by 19% [2]. 

Agro-industrial and food processing by-products and waste are not only a sustainability problem with respect to environmental deterioration, but also an economic problem since they have a direct impact on production profitability. To improve the sustainability of agro-food production, it is essential to have a comprehensive understanding of the various sources of residual biomass generated throughout the supply and production chains from the farm to the consumer’s table. 

Several studies have focused on the quantitative estimation of residual biomass streams for the recovery of raw materials, chemicals and energy [3,4,5,6]. In addition, the European Waste Catalogue (EWC) [7], provided a standardized description of different by-products/wastes which were identified and classified into several categories according to their production, transportation, handling or treatment.

**Figure 1:**
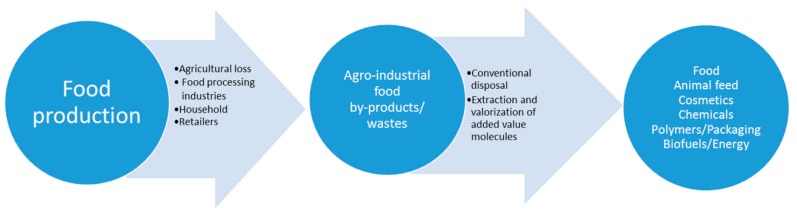
Valorization routes of residues generated by the agro-industrial food processing pipeline.

In general, agro-food by-products and wastes are considered to have little value and are often employed as useful substrates for bioenergy/biofuel production, such as a fermentation substrate for the production of biogas and bioethanol [8,9,10], or as an animal feed, since they meet the minimum quality criteria [11]. Nonetheless, in recent years, agro-food residues valorization practices have attracted significant attention with the aim of finding more sustainable managing systems. In particular, food by-products/wastes represent largely under-exploited residues from which a variety of chemicals can be derived. In fact, these streams could be used more efficiently by developing industrial biorefinery processes aimed at the energy-efficient recovery of high-value components finding application in several industrial markets, as well as at the concomitant production of fertilizers and/or energy [12,13,14,15,16] (Figure 1).

The recovery of valuable compounds from agro-food waste was increasingly investigated and different approaches were considered following the Five-Stage Universal Recovery Process [17]. During this process, in order to effectively separate the targeted compounds from the waste matrix, a progressive separation procedure from the macroscopic to macromolecular, and then to the micromolecular level is applied. Generally, five distinct stages were identified: macroscopic pre-treatment; macro- and micro-molecules separation; extraction; isolation and purification; product formation. Each step can be carried out with different conventional or emerging technologies and the main advantage of this strategy is that it can be applied for simultaneous recovery of several ingredients in different streams [17,18].

Specifically, legume residues are rich in proteins and peptides as well as fibers that can be extracted and further valorized in several industrial fields [11,19,20,21,22], making culture practices more profitable and reducing human dependency on animal products. In addition, they can also be a potential source of many other bioactive molecules (e.g., phenols, carotenoids, phytosterols and fibers) that have been proven to exert a beneficial impact on human health [23,24]. 

This review article presents an overview of by-product/waste generation during legume agro-industrial processing. These crops, and in particular peas, chickpeas and beans, represent an extremely interesting case due to their steadily increasing production and a high annual turnover in the European Union and worldwide given the rising demand of vegetarian/vegan consumers. Mapping and discussion of the main opportunities related to the extraction and fraction valorization technologies mainly of proteins, fibers and other bioactive molecules (e.g., polyphenols and carotenoids) from these biomass streams are described. Current applications and challenges related to the recovered molecules in the feed, food, cosmetic and packaging sectors are also reported. 

## 2. World and European Legume Production

Worldwide and European total legume production has been increasing in the last ten years by about 34% and 44% respectively, with soybeans, beans, peas and chickpeas being the most abundant crops in terms of total production in 2017 [25] (Table 1).

Overall, the major 2017 legume producers in Europe are France, the UK, Italy, Germany and Spain [25]. Given their continental cold climate, France, Germany and the UK specialized in the cultivation of more productive legume crops (soybean, peas and broad beans), while other countries, such as Italy and Spain, cultivate large areas of a heterogeneous group of leguminous plants more adapted to Mediterranean environments. 

The pea is one of the most important nutritional crops grown across the World and Europe as it is rich in protein content (18%–30% [26]) and can be grown in frost-hardy and cold climates. In general, two types of peas are commonly commercialized: green peas (*Pisum sativum* L.) marketed as fresh or canned, and yellow peas (*Pisum sativum* L. var. macrocarpon) commonly called dry peas as they are marketed in dried form, with yellow peas dominating the global production. According to FAOSTAT food and agriculture database [25], the World production of green peas amounted to 20.70 million tons (MT) in 2017, while the global production of dry peas was estimated at 16.21 MT (Table 1).

**Figure 2:**
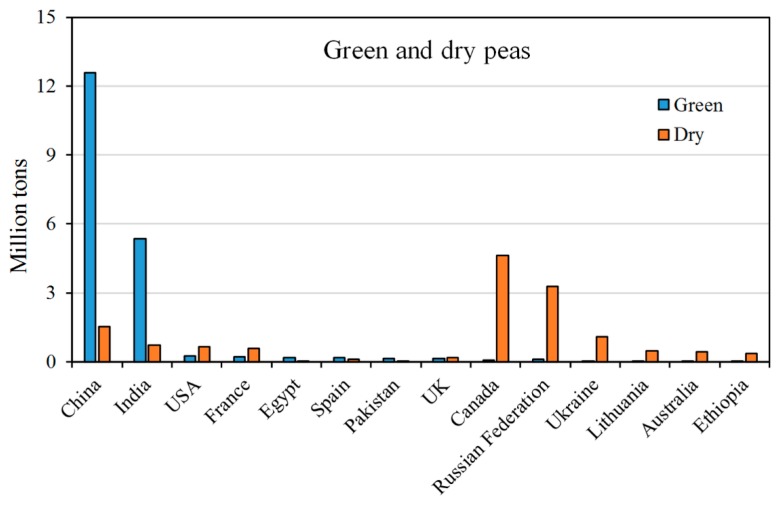
The top world producers (million tons) of green and dry peas in 2017. Data from the FAOSTAT database [25].

**Figure 3:**
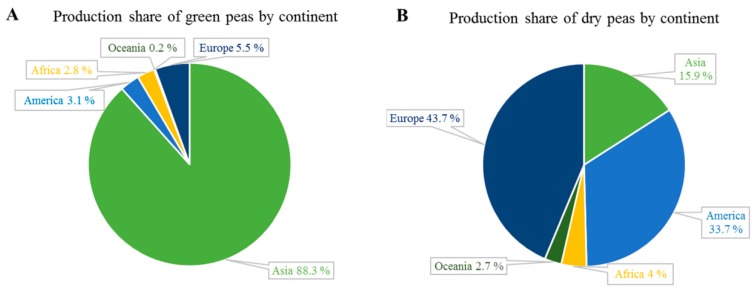
Production share of green and dry peas by region. Data from the FAOSTAT database (year 2017) [25].

In 2017, China was, by far, the country with the largest production of green peas (61% of global production) followed by India (26%) and the USA (1.2%) (Figure 2). Therefore, Asia is the largest green pea producer, covering about 88.3% of global production, while Europe and America, only account for 5.5% and 3.1%, respectively (Figure 3A). In Europe, the major producers are France, Spain and the UK. 

As regards dry yellow peas, in 2017 Canada was the largest producer (4.63 MT), followed by the Russian Federation (3.29 MT) and China (1.52 MT) (Figure 2).

Different from green peas, dry yellow peas are mainly produced in Europe (43.7%), North and South America (33.7%) and Asia (15.9%) (Figure 3B).

The common bean (*Phaseolus vulgaris* L.) is usually known under different names (French bean, kidney bean, snap bean, runner bean or string bean) and is grown and commercialized as fresh seed (green bean) or dry seed. The common bean grows well in medium rainfall areas and it is not suited to the humid and wet tropics.

**Figure 4:**
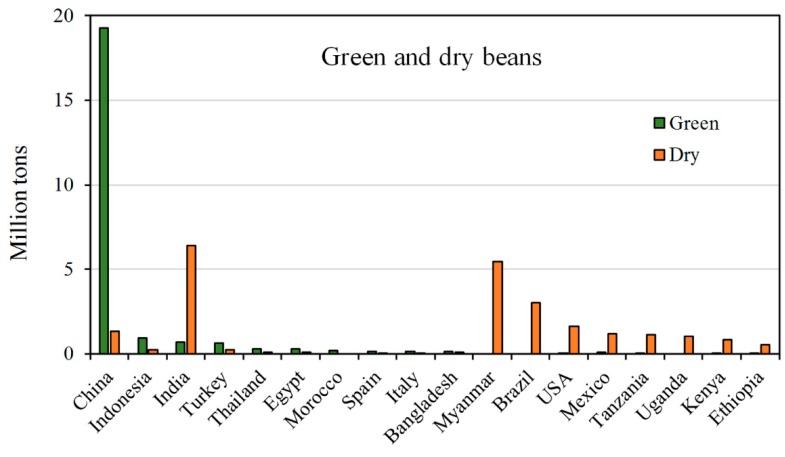
Top world producers (million tons) of green and dry beans in 2017. Data from the FAOSTAT database [25].

The total green and dry bean production reached, worldwide in 2017, a total amount of approximately 55.6 MT (Table 1). The major producer of green beans was China, followed by Indonesia and India, while dry beans were mostly produced by India, Myanmar and Brazil (Figure 4). Europe mostly commercializes green beans (0.77 MT in 2017, Table 1) with Spain and Italy being the countries mainly involved (Figure 4).

Consequently, Asia is the continent with the highest production of both green and dry beans with 91.9% and 49.3% of total production share, respectively. Particularly for dry beans, America and Africa also showed a relevant share in 2017 (25.2% and 21.8%, respectively [25]).

The chickpea is one of the oldest legumes and was domesticated around 3500 BC. The two main cultivated varieties of chickpeas (*Cicer arientinum* L.) are the large, light-seeded Kabuli type, also called garbanzo beans, and the small, dark-seeded Desi type.

The smooth Kabuli chickpeas are mostly farmed in European and African countries surrounding the Mediterranean Sea and are mostly marketed for domestic use. The Desi chickpeas have a rough appearance and a variety of colors, especially in Asian and African countries where they are also sold as milled flour [27]. 

Given its high protein content (almost 40% of seed weight), the chickpea is playing a leading role in covering the deficit in proteins of daily food ratios in Asian (especially Indian) and African sub-Saharan populations. 

**Figure 5:**
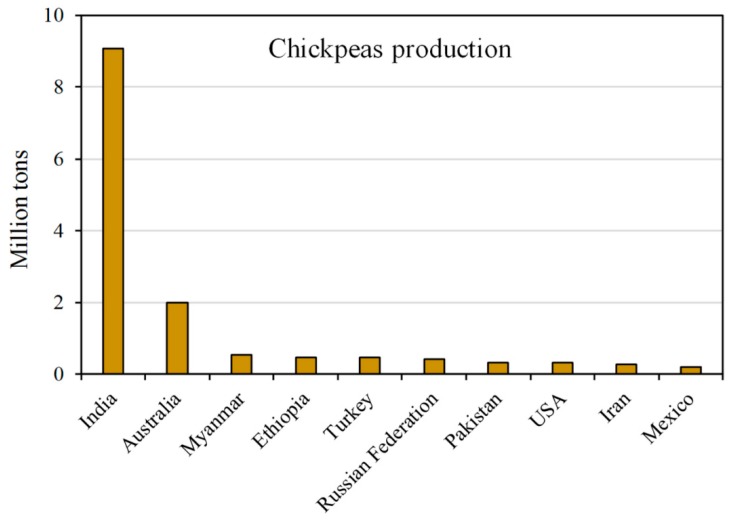
Top world producers (million tons) of chickpeas in 2017. Data from the FAOSTAT database [25].

Globally, India is the largest chickpea producer, accounting alone for about 67% of the total production in 2017 (Figure 5), followed by Australia (14% of share). Europe holds only 4% of the total chickpea production, with Spain (0.06 MT) and Italy (0.03 MT) as major 2017 producers [25]. Worldwide, the chickpea ranks fourth among legume crops, with a production of 14.78 MT (Table 1).

Taken together, the annual combined production of peas, beans and chickpeas accounts for about 65% of global legume production [28] (Table 1).

World and European long-term market trends indicate an increasing demand for high-quality plant proteins. In this view, the growing use of legume proteins seems unavoidable, in particular for peas, beans and chickpeas, due to consumer preference for soy-free products. Therefore, a strong future increment of the world production of these legume crops is foreseen [29,30].

## 3. Legume By-Products/Wastes Generation During the Processing Chain

Throughout the legume agro-industrial processing pipeline, large amounts of residues, by-products and wastes are generated in particular during harvesting and field processing, when damaged legumes are discarded. In addition, pods and other seed residues are left over from the cleaning and splitting operations during industrial processing. 

Pea, bean and chickpea processing (canning, freezing and/or drying) generates a mixture of leaves, stems and empty pods resulting from the fresh legumes processing steps. 

According to estimates from the company Conserve Italia Scarl. (Italy), one of the EU’s largest legume producers and processors, the quantity of residues originating from the legume agro-industrial pipeline ranges from 5% to 25% of the crop initially harvested.

**Figure 6:**
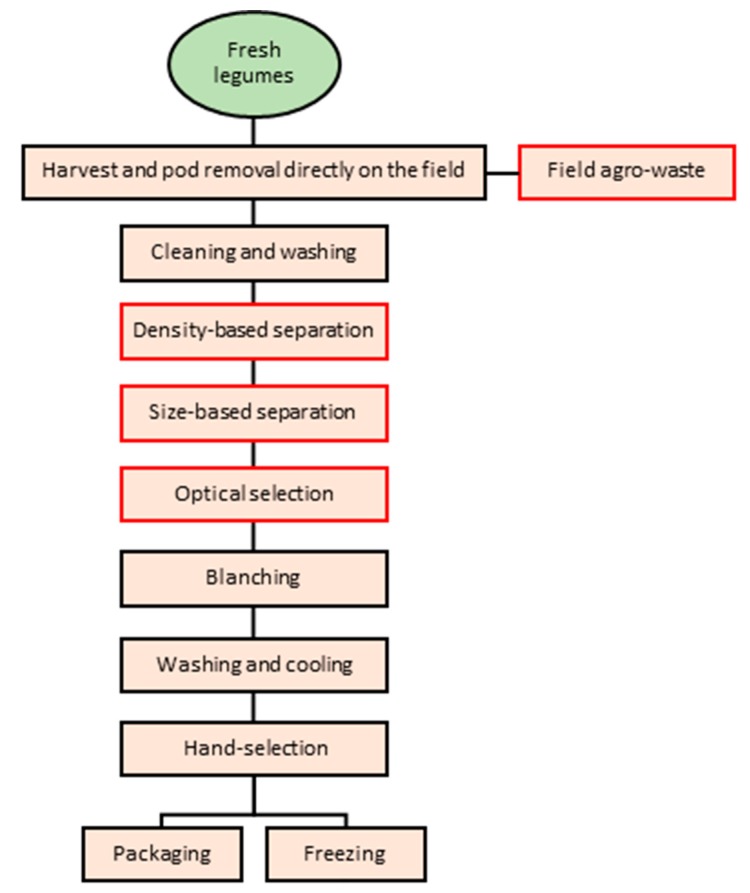
**The** industrial processing scheme of fresh legumes; in red are the steps generating by-products/wastes. Modified from Andreotti [31].

As an example, the flow-chart related to the industrial process of preserved products starting from fresh legumes is shown in Figure 6. In red are the steps corresponding to by-products/wastes production.

**Figure 7:**
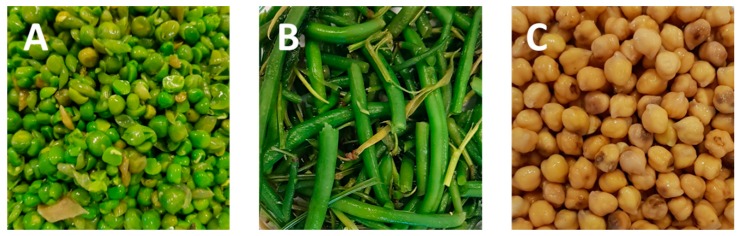
Residues generated during the industrial processing of legumes by Conserve Italia Scarl. (Italy). (**A**) peas; (**B**) green beans; (**C**) chickpeas. Images from Chiara Zurlini.

As shown in the flow chart, after the harvest, the pods are removed directly on the field and shelled generating a large amount of agro-waste consisting of empty pods, leaves and stems. The fresh seeds are delivered to the plant where they enter industrial processing (Figure 6). Generally, considering the transformation processes starting from fresh seeds, by-products are sequentially generated during the initial quality selection steps which start with a density-based separation to select the ripe grains, regardless of the size as legume density usually increases with the degree of maturation [32]. Additional residues are obtained during the size-based separation and the optical selection phases, as well as during the final hand selection before the preserving process [33]. By-products generated during pea industrial processing usually encompass processed and discarded seeds, hulls and dark or spotted seeds (Figure 7).

## 4. Legume Extraction Technologies

Currently, legume extraction and fractionation technologies have been developed mainly to extract proteins and, to a much lesser extent, other molecules (e.g., fibers or phenols) from legume seeds [11,34]. These technologies have been demonstrated to also apply to legume by-products and wastes [19,35,36]. 

Protein extraction can be carried out either through dry or wet processing. Dry fractionation seems to be the most promising technology for protein extraction from legume seeds and it represents a good alternative to water extraction because it preserves protein functionality [34]. The procedure was initially developed for wheat to separate the husk from the grain and wheat germ from endosperm [34]. It consists of fine milling to detach starch granules and protein bodies, thus allowing subsequent separation based on the size or density of these particles by air classification, also involving electrostatic separation processes. Dry fractionation, for separation of protein-rich and starch-rich fractions, was applied on a commercial scale to pulses (i.e., the dry edible part of legume seeds including yellow peas, chickpeas, lentils, beans) and cereals (in particular wheat) [34,37]. To date, only a few studies demonstrated the valorization of legumes by-products generated after dry milling, such as the recovery of nutrition-valuable protein-rich fractions from moth beans (*Vigna aconitifolia*) husks [35].

Wet processing technologies generally provide flours with a higher protein purity than dry processing. Among these technologies, alkaline/acid, solvent and enzymatic extractions and the use of ultrafiltration membranes were the most applied processes to legumes seeds and fractions [38,39,40].

Aqueous alkaline extraction followed by isoelectric precipitation is the most used technique for the extraction of proteins from legume seeds and residues [19].

The process typically involves a milling and/or defatting pre-treatment of the legume feedstocks to remove fiber and fat and decrease the particle size for efficient extraction. Alkaline extraction (pH 8–11) is conventionally employed to improve protein solubility and is generally followed by filtration to remove insoluble carbohydrate material [36,41]. However, the extreme alkaline conditions may alter the functionality and digestibility of the proteins as a result of denaturation, hydrolysis, cross-linking and racemization, as well as the loss of essential amino acids [19].

Legume protein wet extraction can also be obtained with aqueous buffer solutions or under acidic conditions [41]. The solubility of pulse proteins is also high under very low pH (pH < 4.0); after filtration, the liquid extract is subjected to isoelectric precipitation, cryo-precipitation or membrane filtration to isolate proteins [41,42]. Membrane separation takes advantage of the higher molecular weight of proteins to separate them from other soluble components in the extract. Isolated proteins are then washed and dried to obtain concentrates or isolates, depending on targeted protein purity [41]. This technique is also economically sustainable when applied on a large scale for the production of proteins from different waste sources [43]. 

Membrane-based ultrafiltration (UF) is another important alternative method for legume protein isolation to traditional isoelectric precipitation [41,44,45]. In comparison to the isoelectric approach, this process can be operated under milder conditions and shows a high yield of protein recovery given the efficient membrane retention [19,41,45].

Enzymatic hydrolysis, by using animal or plant-derived proteases under mild conditions (pH 6–8), is also an efficient method that, after optimization of the digestion conditions, does not decrease the functionality of extracted proteins [36,46]. Protein enzymatic extraction can also lead to the production of peptide hydrolysates and may be useful in modulating the biological and functional properties of food proteins. Legume proteins, such as soy [36,47,48], peas and several varieties of beans [49,50] have already been subjected to enzymatic hydrolysis. Protein recovery by enzymatic hydrolysis from agro-food residues, among which legumes and more particularly soybean, has also been widely studied by using different plant or animal proteases, also in combination with carbohydrolases, to promote the solubilization of proteins from cell wall components [19,37,51]. 

Organic solvent (e.g., ethanol) extraction processes have been used on an industrial scale for solid–liquid separation of valuable molecules, such as lipids and proteins, providing final ingredients with good nutritional properties [19,36]. However, during the process, the proteins’ functional properties might be altered, significantly limiting their applications, as shown for proteins extracted from soybeans hulls [52]. Organic solvents can also be used for the extraction of the bioactive phenolic fraction [53] and this technique can be applied to legume by-products [54].

The above-mentioned protein extraction techniques can be assisted by the application of power ultrasound at a frequency of 20 kHz. Ultrasound-assisted extraction (UAE) is an inexpensive and efficient technology to improve the yields achieved using conventional solid–liquid extraction techniques. The main advantages of UAE includes the improved penetration of the solvent into cellular material, enhancement of mass transfer due to the cavitation effect that facilitates the release of extractable compounds [17] and the reduction in the use of hazardous solvents, classifying it as a green technique complying with the standards set by the Environmental Protection Agency (USA) [55]. 

Recent research revealed that UAE intensifies the extraction of valuable components from soybeans leading to 10% improved yields of protein, oil and solids [56]. Furthermore, the development of an ultrasound-assisted method to extract natural antioxidants from the mung bean seed coat was reported [57]. Lafarga et al. [58] have used acoustic energy in the acid/alkaline extraction of proteins from Ganxet beans and observed increased yields of solubilized proteins, especially when the extraction was performed at high sodium hydroxide concentrations (0.3–0.4 M). The high yield that can be obtained in UAE processes is of major interest from an industrial point of view, since the technology is an add on step to an existing process, thus needing minimal infrastructural modifications. 

Microwave-assisted extraction with electromagnetic waves with frequency ranging from 300 MHz to 300 GHz has also been studied in order to increase bioactive molecules extraction yields. The energy for this range of frequencies directly generates heat within the sample, as a consequence of molecule vibration, and enhances the extraction capacities of other combined methods [59]. Soluble proteins were extracted from soybean seeds by applying a laboratory-scale MW-assisted extraction [60]. However, the application of microwaves to protein extraction from legume by-products and from other sources is only scarcely reported. 

## 5. Applications of Peas, Beans and Chickpeas By-Products and Wastes

A summary of the most relevant applications of peas, beans and chickpeas by-products/wastes and/or of their extracts and of the involved bioactive molecules is reported in Table 2. 

### 5.1. Feed

In recent years, increasing human consumption of meat has raised market demand for grain legumes for animal feed, resulting in a massive production of residual legume biomass that needs to be further valorized [61]. 

The increased interest in legume by-product/waste streams lies mainly in the possibility of recovering high-quality proteins, which are characterized by high levels of palatability and digestibility and could be further used as feed for all forms of livestock. This application contributes to the reduction of cereal and soybean levels in livestock diets in intensive production systems. Recently, it was estimated that 10% to 20% of livestock diet consists of various legumes, with a maximal inclusion level of up to 50% (FAOSTAT data, 2017 [25]).

Considerable research on the use of legumes and their by-products as animal feed was carried out by several groups and summarized in the reports published by FAO for the occasion of the International Year of Pulses declared by the United Nations General Assembly in 2016 [62,63]. According to these publications, the potential use of legumes and their by-products as feed is mainly due to two factors: 1) the positive contribution of nutrients to the animal diet; 2) the low presence of anti-nutritional factors. Legumes and their by-products are in fact important for animal nutrition as they are excellent sources of amino acids, carbohydrates, fibers, minerals, vitamins, phenols and essential fatty acids [12,20,21]. In general, legume by-products have higher dry matter digestibility, contain more energy for metabolism and have lower fiber content than cereals. Thus, complementing animal feed with different varieties of legume-derived ingredients, significantly improves animal nutrition [63]. However, legumes also contain various anti-nutritional factors (e.g., lectins, agglutinins, saponins, cyanogenic glucosides, alkaloids and biogenic amines) [64,65], which may affect their direct use as animal feed, particularly in monogastric animals (e.g., poultry) [63]. 

Nevertheless, the effects of these factors disappear or decrease when legumes are properly processed (e.g., by roasting, soaking, cooking, autoclaving, boiling, fermentation and seed de-hulling) [64,65].

Among the different types of legume by-products, in particular empty pea pods and left-overs after pea shelling, were deeply investigated [66,67]. These residues are rich in crude proteins (about 20.4% of dry matter (DM)), fiber (neutral detergent fiber 48.1% DM; acid detergent fiber 35.4% DM), total soluble carbohydrates (35.6% DM), total phenolics (9.4% DM), macrominerals (e.g., 0.85% DM of Ca, 0.38% DM of Mg) and microminerals (e.g., 237 ppm of Fe) [66]. Pea pods could serve as a highly palatable source of nutrients for ruminants and could help to decrease the costs in animal farming [66]. 

Several by-products of chickpea cultivation and processing are used for animal feeding, including low-grade and culled chickpeas, bran from de-hulling, crop residues (husks, straw) and chickpea hay [76]. In particular, chickpea straw contains higher nutritive value than cereal straws (44%–46% total digestible nutrients and 4.5%–6.5% protein, DM basis) and is more palatable than wheat straw. Therefore, it can be used as a ruminant feed [77]. Compared with other straws, chickpea straw has a relatively high nutritive value (e.g., metabolizable energy = 7.7 MJ/kg DM for chickpea straw vs. 5.6 MJ/kg DM for wheat) [77,86], but lower than that of other legume straw such as broad bean (*Vicia faba* L.), lentil (*Lens culinaris* Medik) or pea [86]. 

Dry matter digestibility and rumen degradability of chickpea straw were 10% to 42% higher than in cereal straws, indicating that they can be used as an alternative forage in ruminant diets [77]. However, even though its nutritional characteristics are similar to those of other important grain legumes such as pea, chickpea is less used in animal feeding [77].

### 5.2. Food

Nowadays, there has been a growing interest in the food industry towards the potential utilization of legume by-products, mainly related to the presence of high amounts of proteins which could be exploited to create meat analogs for vegetarian/vegan diets, and more generally in the formulation of functional food for human consumption [87,88]. Vegetarian and vegan diets have in fact become more and more popular and many consumers see themselves as partial vegetarians and greatly restrict their consumption of animal products. This has led to an increase in the demand for alternative sources of food proteins mainly coming from legumes and their processed products. Proteins enhance the feeling of satiety, can contribute to lowering blood pressure and have a favorable effect on lipid metabolism. For this reason, legume flours and extracts are important sources of plant protein [89]. Legume protein flours, concentrates and isolates can be incorporated into various types of foods (e.g., high protein pasta, crisps, burger patties, nuggets, beverages, baby food, imitation cheese, whipped toppings, soy milk and baked products) to increase their nutritional value and/or to provide specific and desirable functional or technological properties [41,88,90,91,92]. Flours from different types of legume by-products (*Cajanus cajan*, *Phaseolus aureus* Roxb., *Phaseolus mungo* Roxb.) have been produced and tested to formulate deep-fried snacks, which were evaluated for their physico-chemical, shelf-life and sensory properties [71]. In addition, flours of pigeon pea by-products have been used to produce biscuits with high protein and 10% lower wheat flower contents [68].

Legume proteins, also extracted from legume residual feedstocks, have been hydrolyzed using economically valuable techniques (e.g., physical, chemical or enzymatic digestion) to produce bioactive peptides [49,51,93]. These peptides showed several potential biological activities, such as angiotensin converting enzyme (ACE)-inhibitory and antioxidant activities [49,93], making them interesting as functional ingredients for food or cosmetic applications. 

In recent years, beside vegan and vegetarian products, also the demand of functional food fortified with plant-derived bioactive compounds is progressively increasing. New functional food demand is driven by consumers that are highly aware of the close relationship between nutrition and health and want to include food added with health-promoting ingredients (e.g., fibers and fatty acids) in their diets. Several studies have therefore focused on the development of new ingredients with improved nutritional profiles also recovered from agro-industrial by-products [94]. Among these, legume residues are rich in dietary fibers and, in particular, broad beans and pea pods were studied as a potential nutritionally valuable and significant source of dietary fiber (above 50% of total weight) for human food consumption [20,21]. Chickpea husks have been studied as a source of dietary fibers showing that their addition to baked products, such as white bread, could produce health benefits [80]. Analogously, chickpea hulls also proved to be a good alternative source of dietary fibers and antioxidant phenolics exploitable as ingredients in functional food products. In addition, a wide variety of polyphenols and other molecules (e.g., peptides and lectins) with antioxidant, antimicrobial and other beneficial activities have been extracted from legume residues [21,53,95]. These compounds have the potential to answer the needs of the food industry by introducing alternative antimicrobial compounds from natural sources and by formulating new green-labeled products. For example, the antibacterial and antifungal properties of chickpeas and peas were studied in relation to their protein and peptide profiles [95].

A great variety of natural sources have been investigated for their antioxidant potential in meat and meat products [96]. Natural antioxidants have a higher consumer acceptance and therefore show a larger range of potential applications in the meat industry, with respect to synthetic molecules [78]. Among many others, also legume phenols, such as some flavonoids showing antioxidant and anti-nitrosant activities coming from chickpea, pigeon pea and mung bean [79], may find an application as additives in meat food products where they take part in the prevention of meat oxidation, which is an important cause for off-flavoring [78]. In addition, peroxidation of meat fats or N nitrosation, were found among the main causes of statistically significant dose-response relationship between colon-rectal cancer and the consumption of red meat [97]. To this extent, regarding legume extract application, soy protein hydrolysates were added to cooked ground beef with the aim of reducing lipid oxidation [98]. As excess fat may be a pro-cancer factor, fat reduction by replacement with soluble fibers and/or with other protein-rich ingredients (such as chickpea protein-rich flour) seems to be one of the solutions to make healthier processed meats with better nutritional profiles, but without modifying texture and flavor [99]. 

A particular and interesting legume by-product is aquafaba, the viscous water resulting from chickpea seeds processing, including both the liquid from canned legumes as well as the boiling water from industrial production [83]. It is composed of carbohydrates, proteins, and other soluble plant solids that have migrated from the seeds to the water during the cooking process. The combination of the previous compounds provides this liquid with a wide spectrum of emulsifying, foaming, gelatinizing and thickening properties. Aquafaba is currently used to a limited extent by the food industry, mainly as a substitute for egg white in mayonnaise, dairy products, baked goods (such as meringues and sponge cakes), vegetable snacks, salad dressings and other foods, whereas it has become a popular alternative among vegans [83,84]. 

### 5.3. Cosmetics

There are many commercial applications of legume extracts for cosmetics but none of them are derived from by-products/wastes. Dried seed legume protein fractions and pea peptide hydrolysates are used as skin moisturizers and soothing/anti-itching actives [100,101]. Legume proteins also showed properties for reducing body perspiration and high fiber pea flour was used as an emulsifier [102]. A commercially available pea extract under the trade name ACTIWHITE PW LS 9860 (by BASF, Germany) is currently used as a skin whitening agent [103]. 

According to previous reported studies, bioactive protein/peptides, fibers, polyphenols and other molecules could be successfully extracted from legume processing residues [20,21,67] in view of their application as cosmetic ingredients, similarly to phenolic extracts obtained from other types of agro-waste residues [104]. 

### 5.4. Packaging

Recent trends aim at recovering valuable molecules from legume agro-waste and by-products to be used in the formulation of polymeric materials with new functionalities [105,106]. The packaging sector is looking for solutions to modify bio-based and biodegradable polymers in order to meet challenging requirements for food and cosmetics preservation while maintaining their sustainability and biodegradability. One of the main goals is to reduce the consumption of highly expensive bio-based and compostable or biodegradable polymers (such as polyhydroxyalkanoates (PHA), polybutylene succinate (PBS), polycaprolactone (PCL), polylactic acid (PLA)) as well as impart to the biodegradable materials the same properties of the fossil-based ones. In this context, the formulation of materials including residues and/or bioactive molecules extracted from natural wastes/by-products was promoted [107]. Technologies and procedures applied to recover proteins and active compounds from legume and other plant feedstocks, also generate a considerable amount of fiber and residual biomass. These can then be valorized for the production of bio-composite materials obtained by mixing a biopolymeric matrix (e.g., PHA, PLA, PBS and PCL) and a defined percentage of reinforcing bio-fillers, such as plant-derived fibers. As natural fibers are renewable, biodegradable, available in large amounts and cheap, they are particularly interesting for the preparation of polymer composites where they can improve the mechanical properties of the matrices and reduce the amount of expensive bio-polymeric matrix present in the final material [108,109]. The residues remaining after protein extraction from legumes have been exploited for the preparation of bio-composites together with polyhydroxyalkanoates (PHAs) as a biodegradable polymeric matrix [110]. This approach enabled the production of rigid packaging items with good mechanical properties and opened perspectives for a packaging market based on new sustainable solutions. In addition, the production of aluminum matrix composites (AMCs) using bean pod ash nanoparticles was studied for automobile application, giving good and promising results [72]. In fact, bean pod ashes can be considered as a promising reinforcement for the production of biocomposites, due to their low density, low cost and availability in large quantities as agricultural wastes. 

Aromatic amino acids (such as tryptophan and tyrosine) obtained from the hydrolysis of a protein-rich agricultural waste can also be utilized to impart high UV resistance and UV-shielding features to a polymeric matrix [111]. However, embedding these molecules directly into a polymeric matrix has several drawbacks since they can react with the polymer back-bone giving rise to polymer degradation, or they can leach out of the polymer surface itself. In order to overcome these drawbacks, a protection strategy using hydrotalcite-like compounds (layered double hydroxides, LDH) has been developed [112] to immobilize the amino acids and using them as fillers to impart UV resistance to the polymer matrix [111]. In addition, bio-based polymers and bio-films obtained also from legume seed proteins were produced to be applied as packaging materials and composites. The most studied of these polymers is sourced from the soybean, but films derived from pea proteins showed interesting UV light transmission resistance due to the presence of aromatic amino acids [113]. Given the presence of high amino acid levels in extracted legume agro-waste, it is therefore possible to hypothesize a possible extension of aforementioned applications to protein extracts coming from legume processing residues.

Furthermore, an interesting and recent application in the packaging field is the development of a new type of bioplastic based on chickpea aquafaba [85] that shows great potential for mechanical manufacturing and thus industrial production being completely biodegradable and also vegan-friendly.

Finally, a 100% recyclable packaging paper was obtained from bean processing waste by an eco-sustainable process and certified for application in direct contact with food [73].

This type of paper was estimated to reduce the use of virgin cellulose from trees by 15% and the emission of greenhouse gases by 20%. 

### 5.5. Other Uses

Recently, chickpea husk was used to extract textile grade dye that is able to impart color to cotton, silk and wool fabrics as well as to give functional finishing features of textiles by using a totally eco-friendly process [81]. Treated fabrics showed a good dye uptake and adequate wash, light and rubbing fastness properties, in addition to a good ultraviolet protection property and excellent resistance against *Staphylococcus aureus* and *Escherichia coli* bacteria. 

Legume waste was also used in the composting field. Bean dregs (at 0%, 35% and 45%) were evaluated as additives during the two-stage composting of green waste, improving process conditions and compost quality [74].

Other applications are reported in Table 2.

## 6. Conclusions

Legume residues, such as pea, bean and chickpea by-products/wastes, have a high and proven potential in boosting new and diversified market opportunities in several industrial sectors. In particular, the feed and food industries could find in legume residues a valuable source of bioactive and highly nutritional ingredients (e.g., proteins and fibers) that may be obtained by means of green extraction processes from sustainable natural resources. Moreover, the emerging packaging sector is steadily seeking vegetal feedstocks to produce new bio-based materials with improved technical and mechanical features for a wide array of applications, aiming at a progressive reduction of the use of petrol-based polymers and of the cost of the final materials. 

The efficient utilisation of the all agro-industrial residues can, therefore, help in reducing the overall economic and environmental impact of the agro-industrial and food processing pipelines. The residues extraction and following exploitation must, therefore, be carried out with low environmental impact and green technologies, which will lead to achieving, hopefully in the near future, a zero-waste economy and a more sustainable bio-based and circular society.

## Figures and Tables

**Table 1:** European production amount (in million tons, MT) of different legumes. Data from the FAOSTAT food and agriculture database and related to 2017 [25].

Legume Type	World (MT)	Europe (MT)
Beans (dry)	31.41	0.62
Beans (green)	24.22	0.77
Broad Beans (dry)	4.84	0.97
Caw Peas	7.41	0.002
Chickpeas	14.78	0.13
Lentils	7.59	0.07
Lupins	1.61	0.25
Peas (dry)	16.21	2.60
Peas (green)	20.70	0.93
Soybeans	35.26	2.67

**Table 2:** Summary of most relevant applications of peas, beans and chickpeas byproducts/wastes and/or of their derived extracts.

Legume Feedstock	Field of Application	Application	Bioactive Compounds	Outcome	Reference
Pea pods Pulses by-products	Feed	Monogastric and polygastric animal feed	Proteins, fibers, minerals	Biochemical and nutritional characterization. Impact on animal performance.	[62,63,66,67]
Pigeon pea by-products	Food	High protein biscuits	Proteins	Chemical composition; physical and sensory parameters	[68]
Pea and broad bean pods	Food	Food ingredients	Fibers, soluble sugars, minerals, linoleic acid	Biochemical and nutritional characterization; antioxidant activity	[20,21]
Pea pod waste	Bio-resources	Bio-butanol production	Cellulose/hemicellulose	Potential carbon source for bio-butanol production	[69]
Pea peel waste	Bio-resources	Cellulase enzyme production	Cellulose	Potential source for cellulose production	[70]
Moth bean milling residues	Food	Food ingredients	High essential amino acids, fatty acids, minerals.	Water and oil absorption capacities, foaming and emulsification properties.	[35]
Black gram (*Vigna mungo*) milling by-products	Food	Food ingredients	Phenolic acids like gallic, protocatechuic, gentisic, vanillic, syringic, caffeic and ferulic acids	Biochemical and nutritional characterization; α-glucosidase inhibitory activities correlated to potential antioxidant and anti-diabetic properties.	[54]
Red, green and black gram by-products	Food	Deep-fried snacks	Proteins	Sensory results and shelf life studies	[71]
Bean pod ash nanoparticles	Automobile application	Composites with bioreinforcements	Nano-fibers, cellulose	Increased tensile strength and hardness values, reduced weight and energy impact	[72]
Process bean waste	Packaging	Ecopaper for food packaging	Fibers, cellulose	100% recyclable packaging paper obtained by an eco-sustainable process and certified for application in direct contact with food	[73]
Bean dregs	Compost	Compost product of high-quality	Cellulose, hemicellulose	Improved composting conditions and compost quality	[74].
Bean dregs	Bio-resources	Production of reducing sugar	Sugars	Efficient method for biomass wastes liquefaction.	[75]
Chickpea straw	Feed	Alternative forage in ruminant diet	Proteins, fibers	High nutritional value, dry matter digestibility, rumen degradability	[76,77]
Chickpea, mung bean, pigeon pea hulls	Food	Meat additives	Phenolics, flavonoids	Antioxidant, antimicrobial, antinitrosant activities	[78,79]
Chickpea husk	Food	Baking additives	Fibers, polyphenols	Calcium content, antioxidant activity and phenolic compounds content slightly improved; increase in shelf life, rheological, physical and sensory parameters.	[80]
Chickpea husk	Textile	Textile grade dye	Flavonoids, tannins, terpenoids	Functional finishing features of textiles, good ultraviolet protection, excellent resistance against bacteria.	[81]
Chickpeas hulls	Food	Food additives	Fibers, polyphenols	Source of dietary fiber and phenolics with antioxidant capacity	[82]
Aquafaba	Food	Egg-white substitute in food foam and emulsions.	Proteins, carbohydrates	Foaming and emulsification properties	[83,84]
Aquafaba	Packaging	Bioplastic	Proteins, carbohydrates	Biodegradable bioplastic	[85]
